# Supplementary material for: Mannosylated Polymeric Ligands for Targeted Delivery of Antibacterials and Their Adjuvants to Macrophages for the Enhancement of the Drug Efficiency
Source: Pharmaceuticals (Basel). 2022 Sep 21;15(10):1172. doi: 10.3390/ph15101172 (PMC9607288; doi:10.3390/ph15101172)

Supplementary Materials

Mannosylated polymeric ligands for targeted delivery of antibacterials and their adjuvants to macrophages for the enhancement of the drug efficiency

Igor D. Zlotnikov [1,*], Alexander A. Ezhov [2], Rostislav A. Petrov [1], Maksim A. Vigovskiy [3,4], Olga A. Grigorieva [3,4], Natalya G. Belogurova [1] and Elena V. Kudryashova [1,*]

[1] Faculty of Chemistry, Lomonosov Moscow State University, Leninskie Gory, 1/3, 119991 Moscow, Russia;

[2] Faculty of Physics, Lomonosov Moscow State University, Leninskie Gory, 1/2, 119991 Moscow, Russia;

[3] Institute for Regenerative Medicine, Medical Research and Education Center, Lomonosov Moscow State University, 27/10, Lomonosovsky Ave., 119192, Moscow, Russia

[4] Faculty of Medicine, Lomonosov Moscow State University, 27/1, Lomonosovsky Ave., 119192 Moscow, Russia;

* Correspondence: helenakoudriachova@yandex.ru (E.V.K.), zlotnikovid@my.msu.ru (I.D.Z.)

Content

Figure S1. Fourier transform infrared spectra of PEI10-N=CH-triMan and PEI10-triMan. Aqueous solutions. T = 22°C.

Figure S2. Size distribution by volume determined by dynamic light scattering method: (a) amCD-triMan, (b) PEI1.8-triMan, (c) HPCD-PEI1.8-triMan, (d) PEI10-triMan, (e) HPCD-PEI10-triMan. T = 25 °C.

Figure S3. Zeta-potential distribution determined by dynamic light scattering method: (a) amCD-triMan, (b) PEI1.8-triMan, (c) HPCD-PEI1.8-triMan, (d) PEI10-triMan, (e) HPCD-PEI10-triMan. T = 25 °C.

Figure S4. High performance liquid chromatogram of mannosylated drug delivery systems. Knauer chromatography system (Knauer, Berlin, Germany) on Diasfer-110-C18 column (BioChemMack, Moscow, Russia): grains – 6 µm, size 4×150 mm. The eluent was CH_3CN-H_2O (90:10, v:v); the elution rate was 0.8 mL/min, 25 °C.

Figure S5. Fourier transform infrared spectra of ConA and ConA complexes with PEI10-triMan. C_0(ConA subunit) = 0.1 mM. Natrium-phosphate buffer solution (0.02 M, pH 6.4). C(Ca^{2+}) = C(Mn^{2+}) = 1 mM. T = 22 °C.

Figure S6. FTIR spectra of MF and MF complexes with PEI1.8-triMan. 0.01M HCl. T = 22 °C.

Figure S7. Cell viability of *B. subtilis* as a function of incubation time with drug samples. Major active component is MF; adjuvants – EG, menthol and safrole. CFU(0 h) = $8{\cdot}10^6$. pH 7.4 (0.01 M PBS), 32 °C.

Table S1. Confocal laser scanning images of *B. subtilis* with adsorbed and absorbed Dox and FITC-labeled ligands based on PEI, HPCD and triMan. A time comparison (15-30-90 minutes) and dependence on the presence of the adjuvant EG are presented. The scale segment is 50 µm (division value is 10 µm). 3-4 channels are shown: red – Dox, green – FITC, gray – transmission light mode, and overlay.

Figure S8. The dependence of *B. subtilis* colony-forming units (CFU) on the incubation time with **(a)** EG (0.1 mg/mL), **(b)** SDS (0.1%). Medium LB (pH 7.2), 37 °C.

Figure S9. Fourier transform infrared spectra of centrifuged *B. subtilis* followed by resuspension in PBS (0.01 M, pH 7.4): **(a)** cells alone; **(b)** cells hour-incubated with 0.1 mg/mL Dox-MCD and 0.1 mg/mL Dox-MCD with 0.1% SDS. CFU = $3{\cdot}10^6$. T = 22 °C.

Figure S10. Emission fluorescence spectra of FITC and Dox obtaining by CLSM. λ_{em} = 488 nm (multiline Argon laser).

Figure S11. Immunocytochemical evaluation of CD206 in primary culture of human dermal fibroblasts (HDF) (red channel – low intensity). Phagocytosis assay with HPCD-PEI1.8-triMan and HPCD-PEI1.8-Man conjugated with a FITC fluorescent label after incubation for 40 min (green channel). Phase contrast microscopy, fluorescent microscopy, blue channel — nuclei stained with DAPI. Scale bar, 100 µm.

Figure S12. Flow cytometry data on distribution of the number of human dermal fibroblast cells by the intensity of absorbed FITC-labeled conjugates HPCD-PEI1.8-triMan and HPCD-PEI1.8-Man.

Figure S1. Fourier transform infrared spectra of PEI10-N=CH-triMan and PEI10-triMan. Aqueous solutions. T = 22°C.

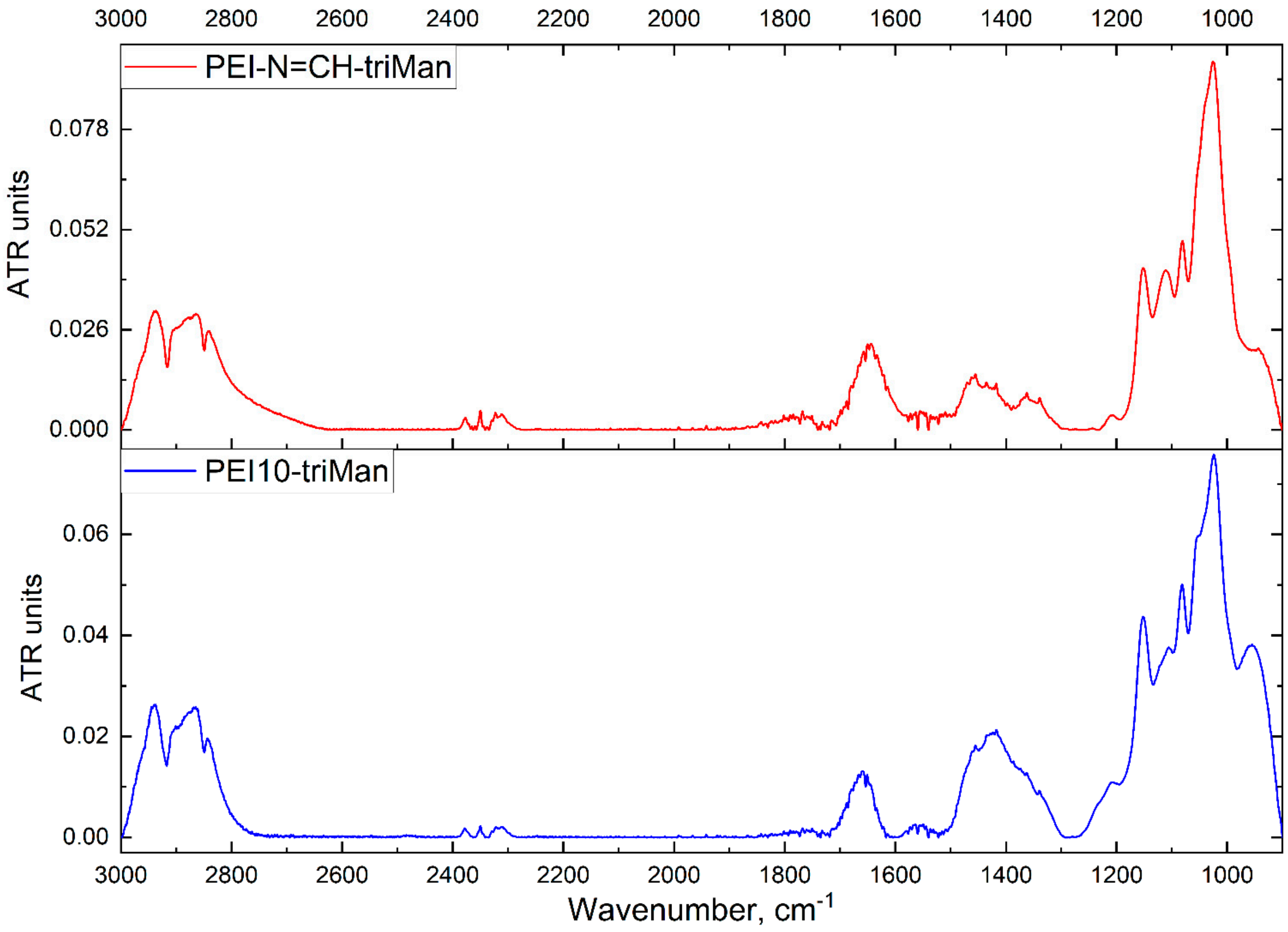

Figure S2. Size distribution by volume determined by dynamic light scattering method: (a) amCD-triMan, (b) PEI1.8-triMan, (c) HPCD-PEI1.8-triMan, (d) PEI10-triMan, (e) HPCD-PEI10-triMan. T = 25 °C.

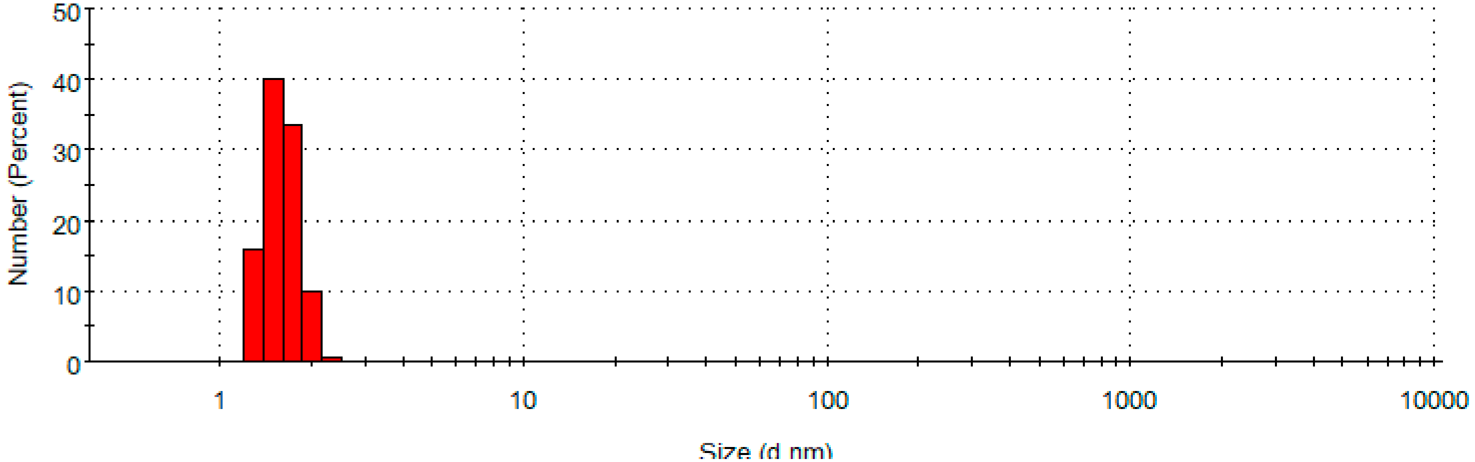

(a)

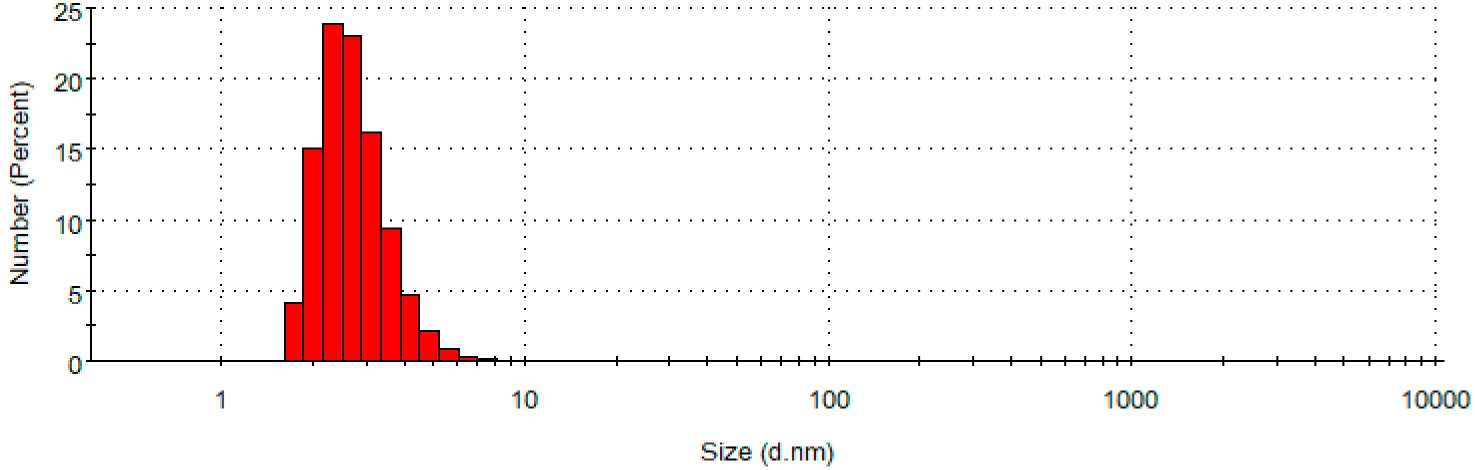

(b)

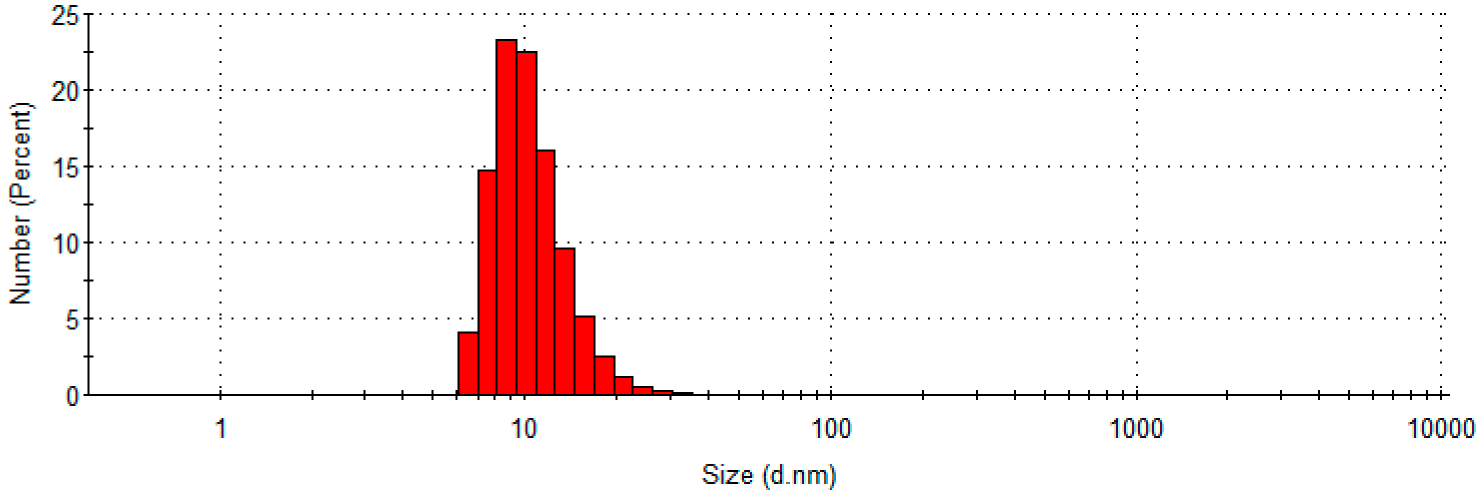

Number (Percent)
25
20
15
10
5
0
1
10
100
1000
10000
Size (d.nm)

(c)

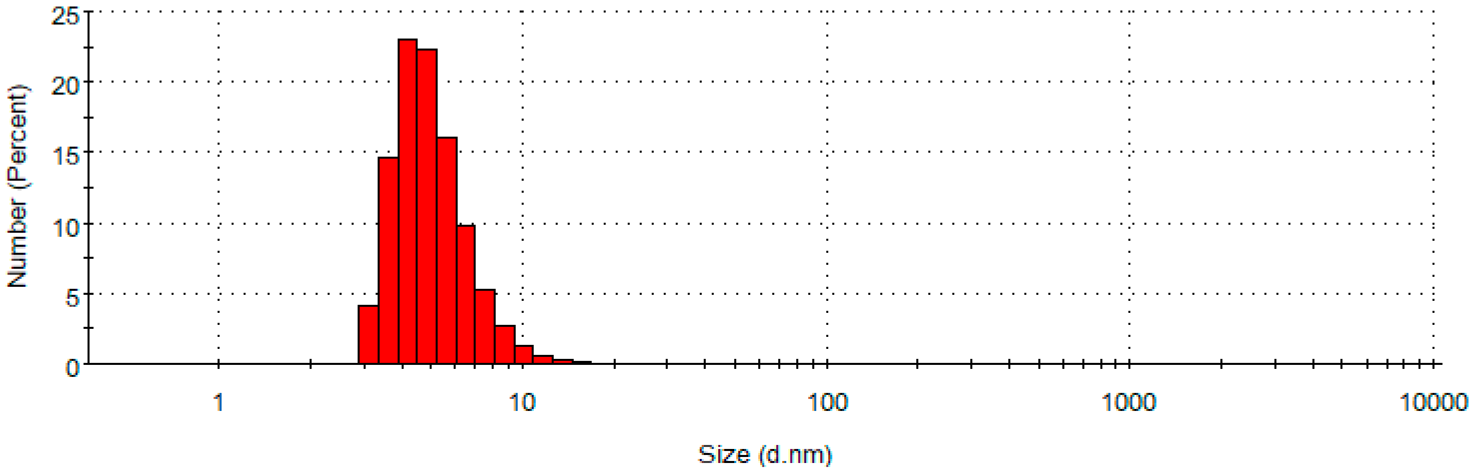

Number (Percent)
25
20
15
10
5
0
1
10
100
1000
10000
Size (d.nm)

(d)

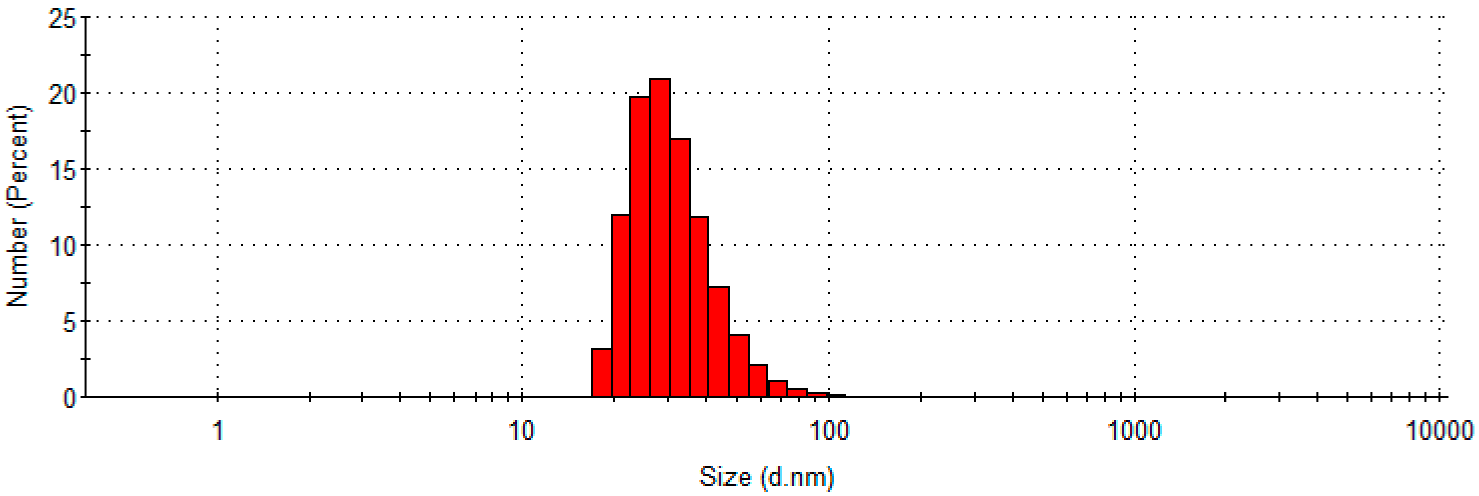

(e)

Figure S3. Zeta-potential distribution determined by dynamic light scattering method: (a) amCD-triMan, (b) PEI1.8-triMan, (c) HPCD-PEI1.8-triMan, (d) PEI10-triMan, (e) HPCD-PEI10-triMan. T = 25 °C.

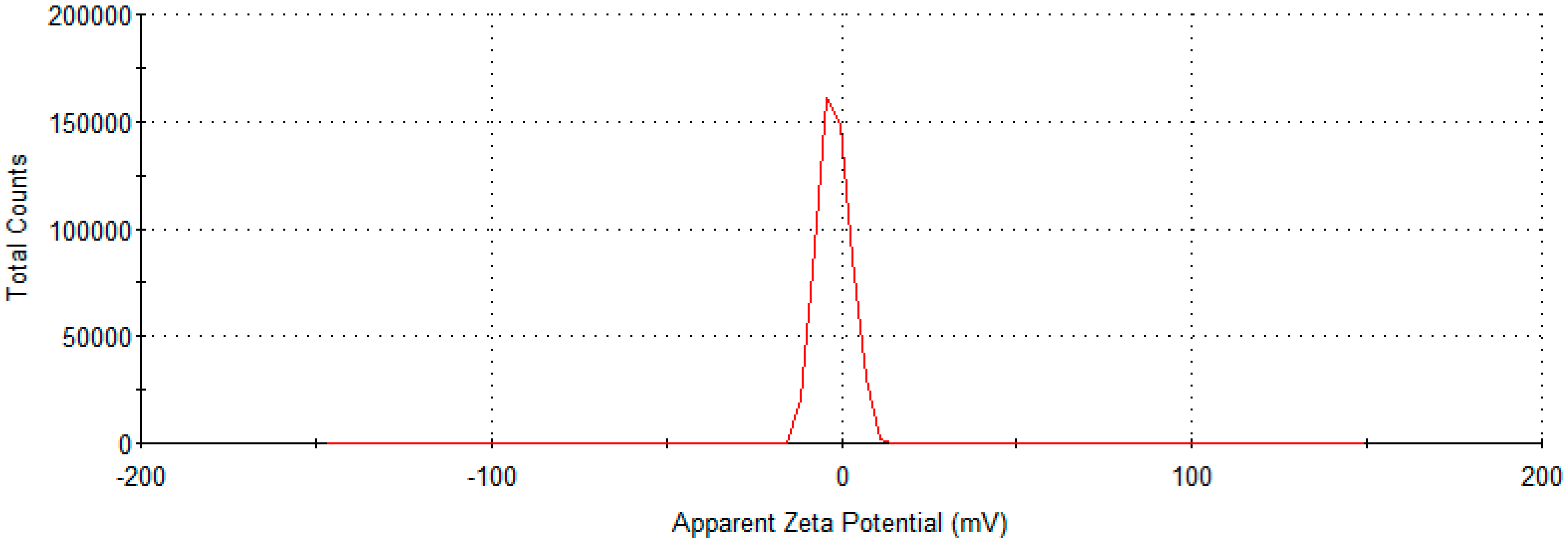

(a)

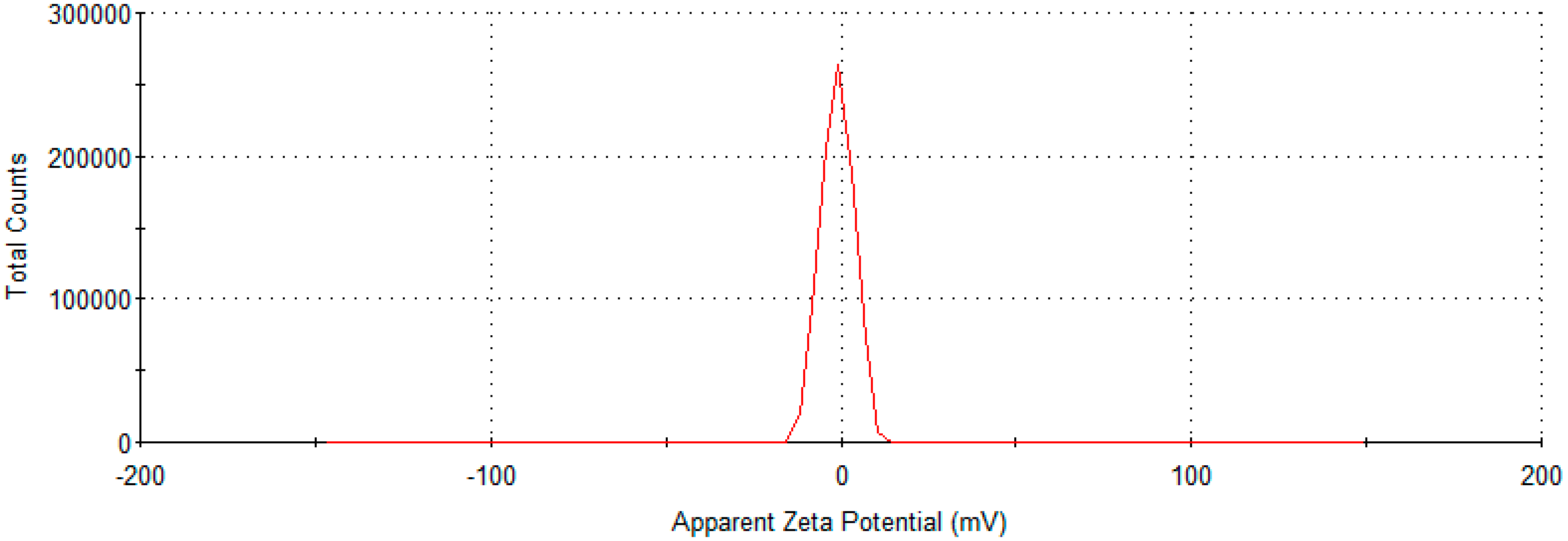

(b)

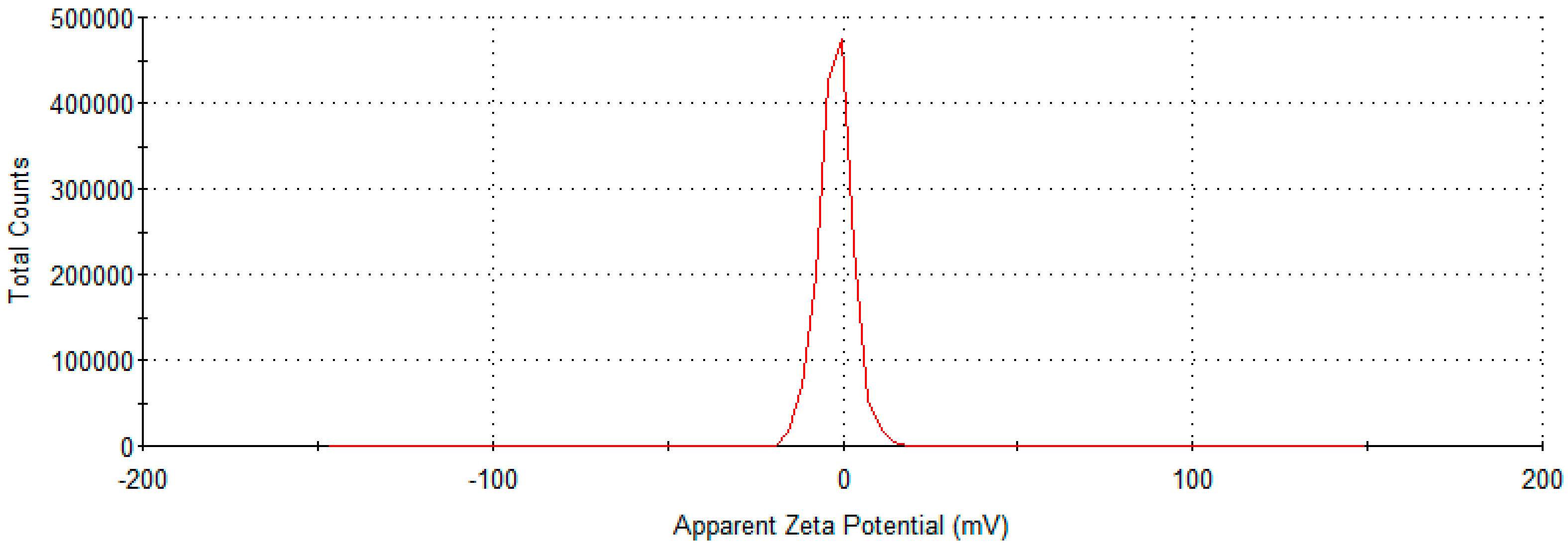

500000
400000
300000
200000
100000
0
Total Counts
-200
-100
0
100
200
Apparent Zeta Potential (mV)

(c)

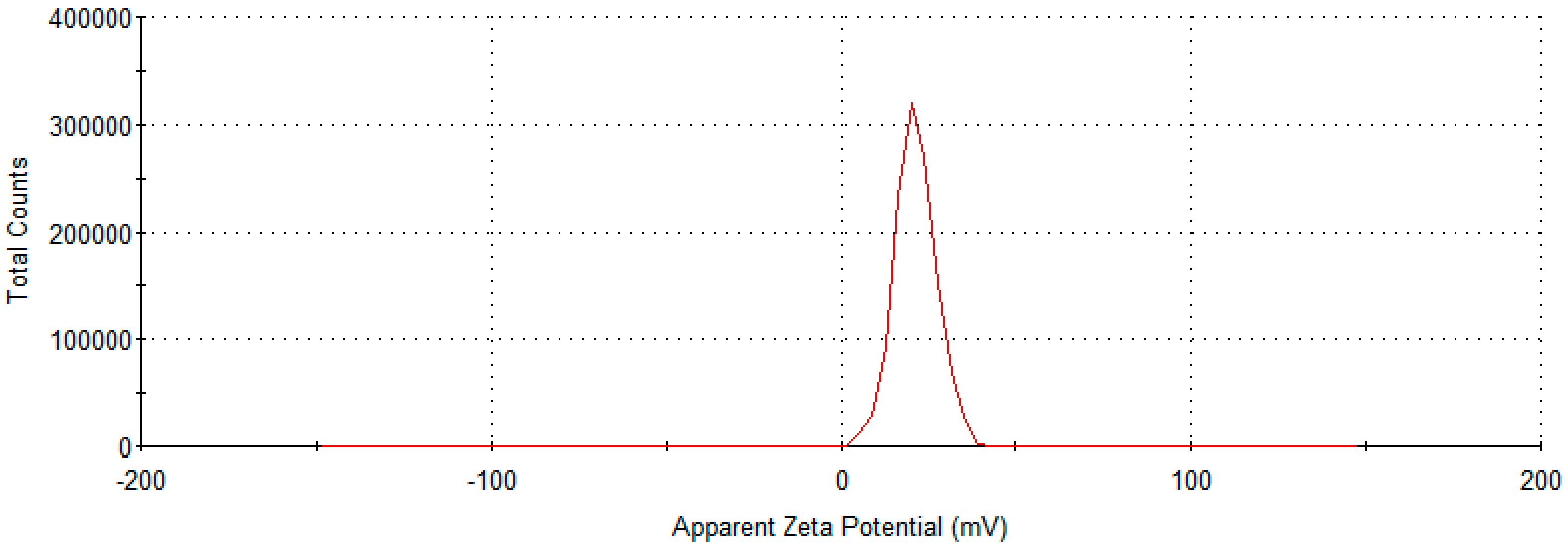

400000
300000
200000
100000
0
Total Counts
-200
-100
0
100
200
Apparent Zeta Potential (mV)

(d)

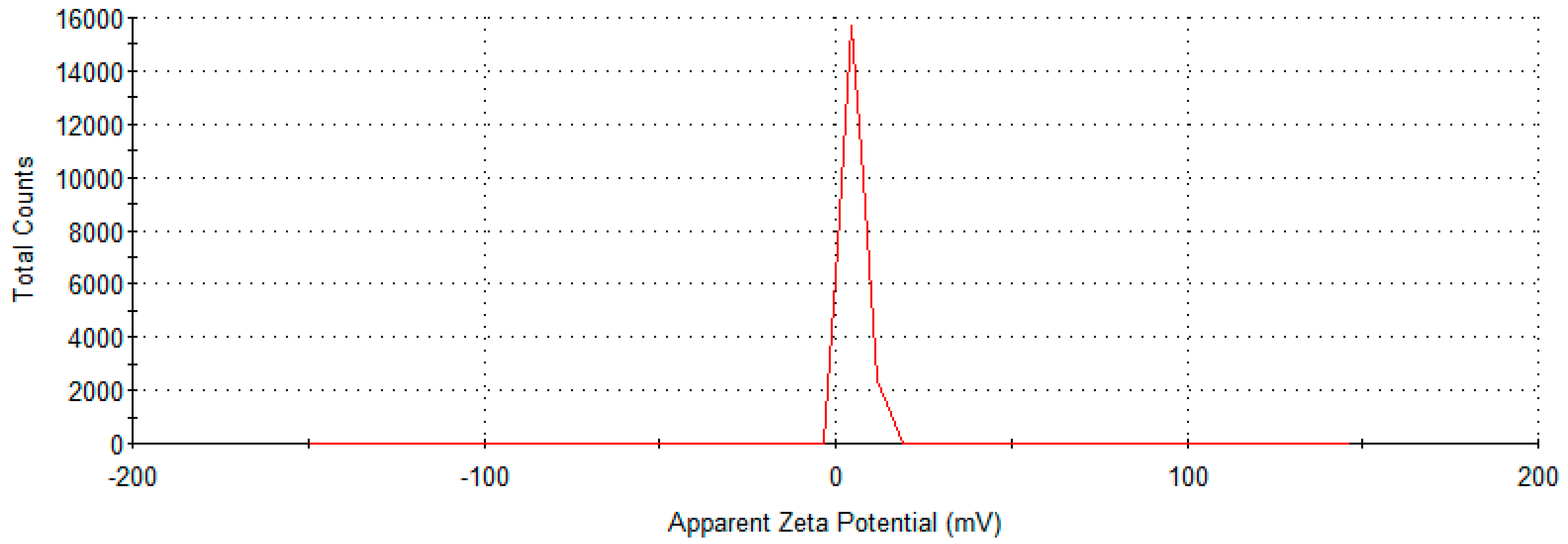

16000
14000
12000
10000
8000
6000
4000
2000
0
Total Counts
-200
-100
0
100
200
Apparent Zeta Potential (mV)

(e)

Figure S4. High performance liquid chromatogram of mannosylated drug delivery systems. Knauer chromatography system (Knauer, Berlin, Germany) on Diasfer-110-C18 column (BioChemMack, Moscow, Russia): grains – 6 µm, size 4×150 mm. The eluent was CH_3CN-H_2O (90:10, v:v); the elution rate was 0.8 mL/min, 25 °C.

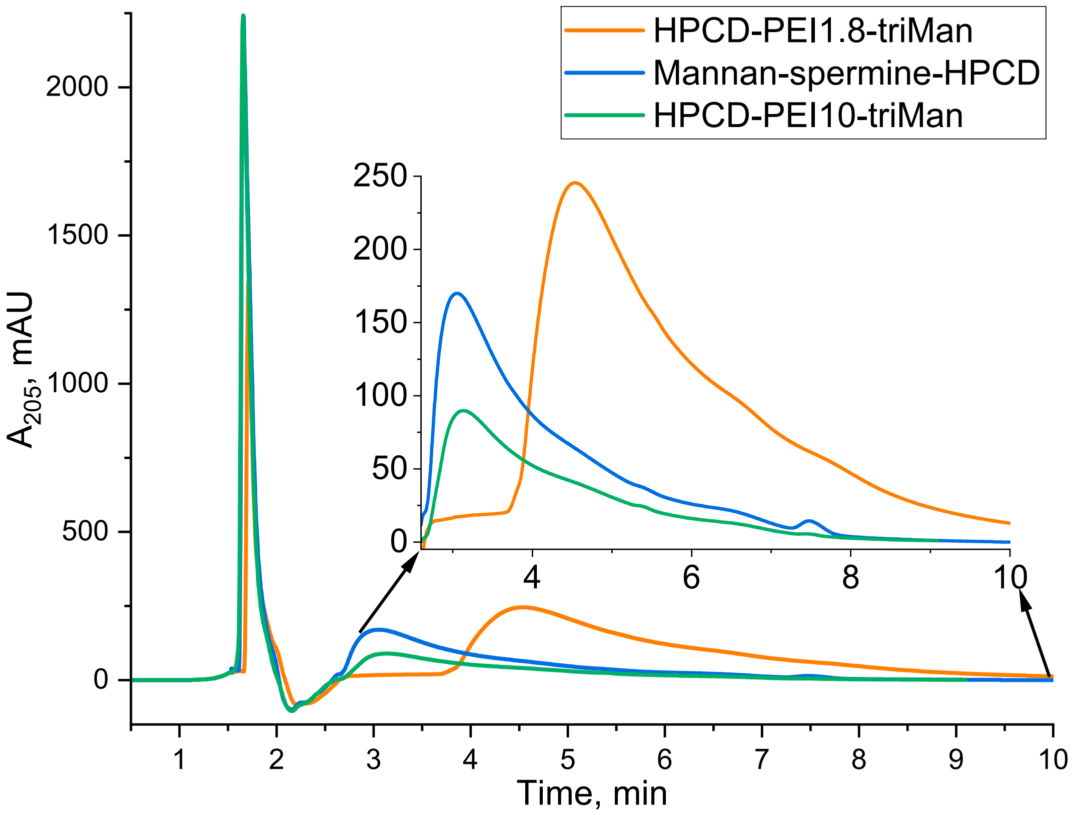

Figure S5. Fourier transform infrared spectra of ConA and ConA complexes with PEI10-triMan. C_0(ConA subunit) = 0.1 mM. Natrium-phosphate buffer solution (0.02 M, pH 6.4). C(Ca^{2+}) = C(Mn^{2+}) = 1 mM. T = 22 °C.

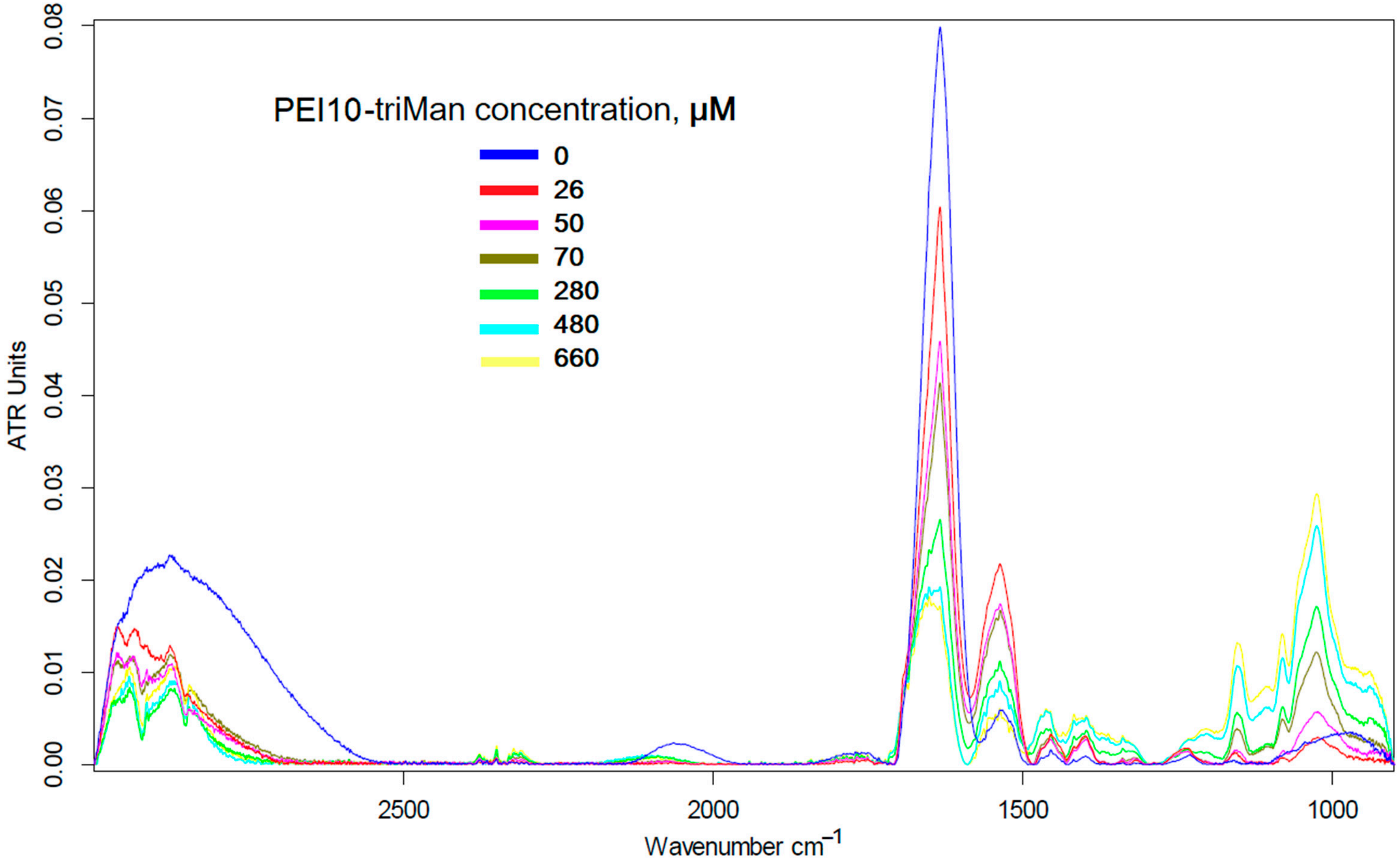

Figure S6. FTIR spectra of MF and MF complexes with PEI1.8-triMan. 0.01M HCl. T = 22 °C.

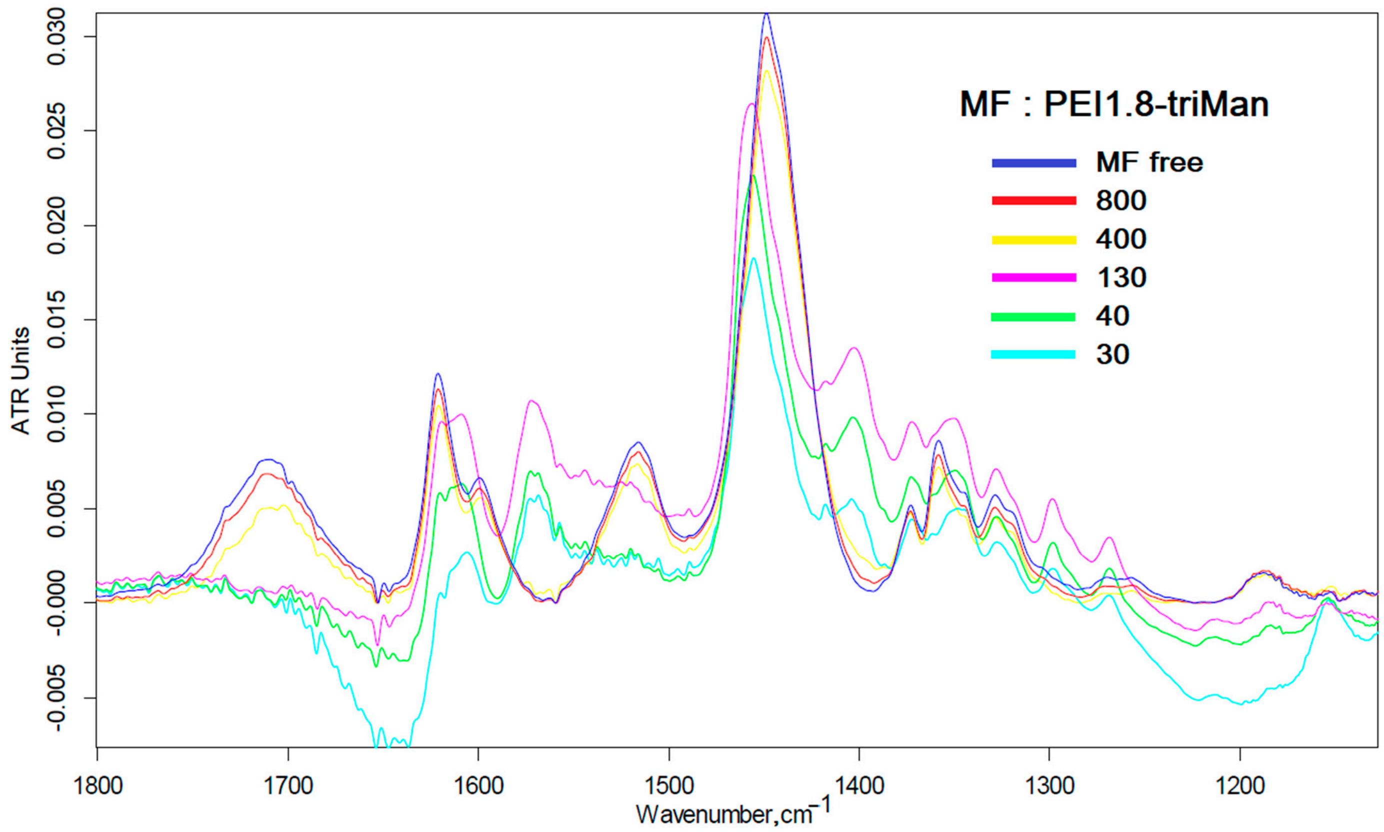

Figure S7. Cell viability of *B. subtilis* as a function of incubation time with drug samples. Major active component is MF; adjuvants – EG, menthol and safrole. CFU(0 h) = $8 \cdot 10^6$. pH 7.4 (0.01 M PBS), 32 °C.

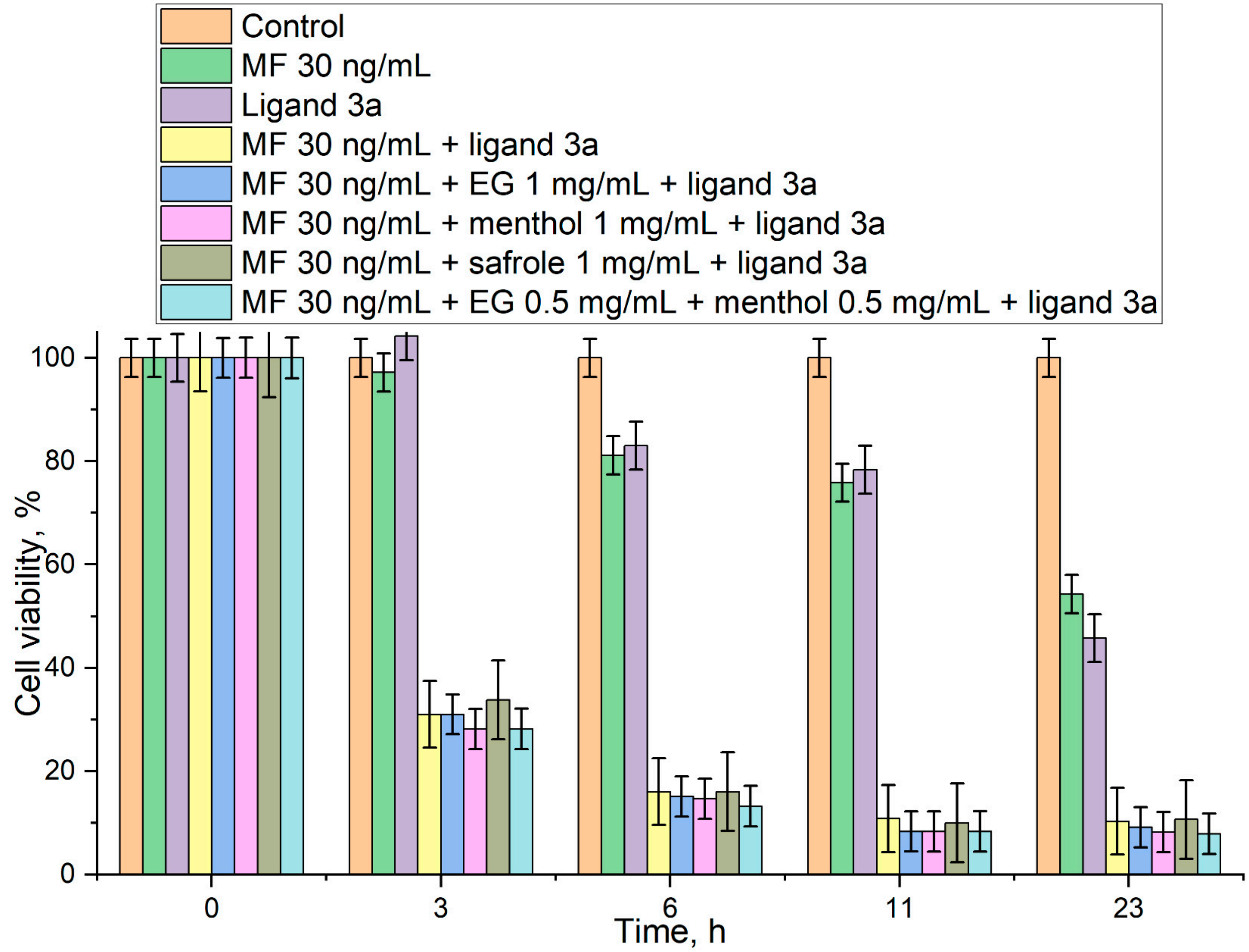

Table S1. Confocal laser scanning images of *B. subtilis* with adsorbed and absorbed Dox and FITC-labeled ligands based on PEI, HPCD and triMan. A time comparison (15-30-90 minutes) and dependence on the presence of the adjuvant EG are presented. The scale segment is 50 μm (division value is 10 μm). 3-4 channels are shown: red – Dox, green – FITC, gray – transmission light mode, and overlay.

Comments:

1) Free Dox accumulates weakly in cells. The maximum concentration among the studied times is after 15 minutes. Over time, Dox is released by pump proteins from the efflux cell. Therefore, the drug practically does not penetrate the cells.
2) The addition of the adjuvant EG to Dox due to the occurrence of defects in the cell membrane and inhibition of pump proteins increases the degree of accumulation of Dox in cells. When EG acts in two directions (creates defects in the membrane and inhibits pump proteins), the effect of enhancing drug penetration increases over time. However, preincubated with cells for an hour and then removed from the solution and adsorbed on the membrane, EG can act primarily as an inhibitor of efflux proteins and to a lesser extent destroy the membrane, which causes a maximum effect after 30 minutes. EG effectively "turned off" the pump proteins and a significant amount of Dox was able to penetrate into the cells.
3) Consider the effect of molecular containers (ligands) on the penetration of Dox. Due to the adsorption of polymer particles on the cell membrane and the resulting defects of the Dox cell wall, the penetration of Dox increases with a time of 0-15-30-90 minutes. Moreover, the greatest effect is achieved for the polymer ligand 2b grafted with CD tori, since Dox is more efficiently loaded into the hydrophobic cavities of the CD. And besides, CD additionally enhances penetration through the membrane.
4) The total effect is observed in the system (Dox + EG + FITC-HPCD-PEI1.8-triMan), in which the penetration of Dox increases due to all of the above effects in synergy with each other. Moreover, the ligand loads both EG and Dox, and since the distribution of components is uniform for such double inclusion complexes, two EG and Dox molecules are likely to be next to each other at once. The first one will create a defect in the membrane through which the target substance, Dox, will penetrate "without wasting time".

Sample	CLS image – 15 min	CLS image – 30 min	CLS image – 90 min
**Dox**	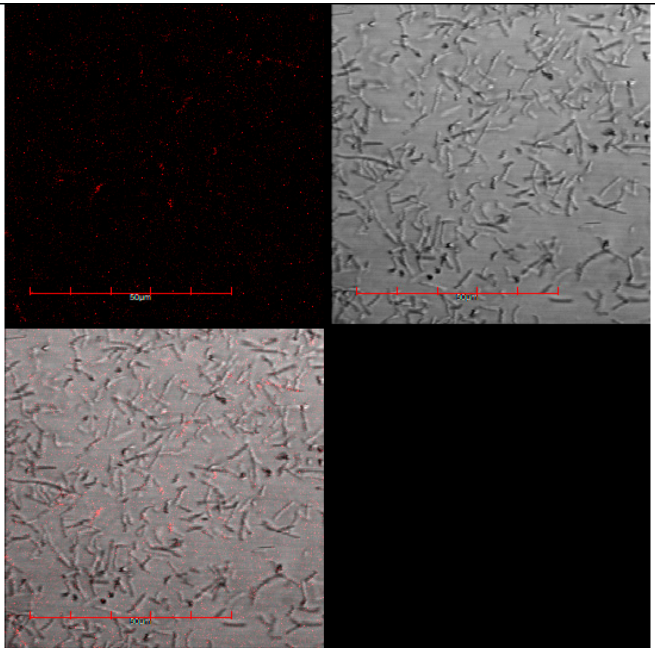	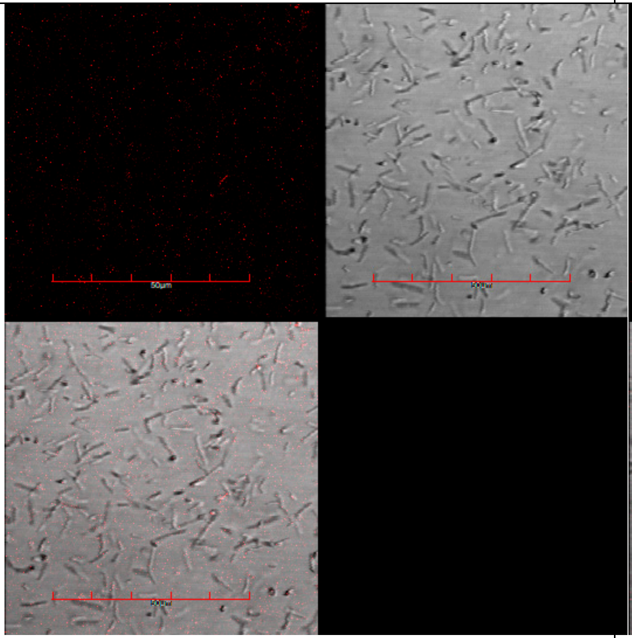	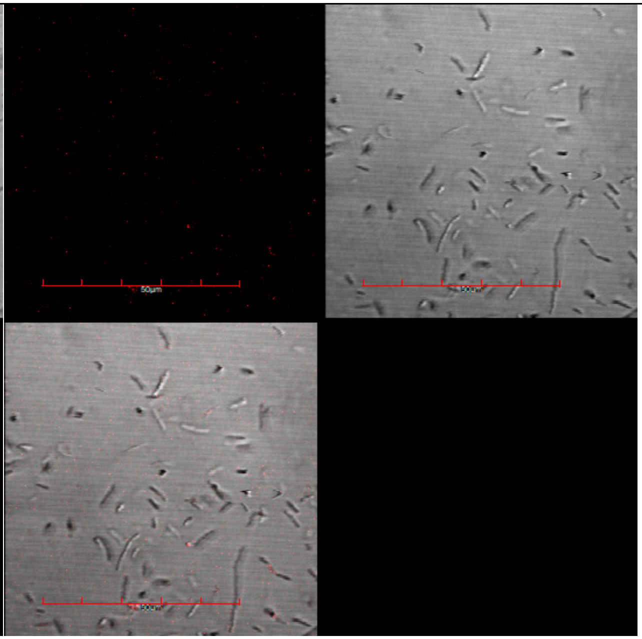
**Dox + EG (membrane defect + efflux)**	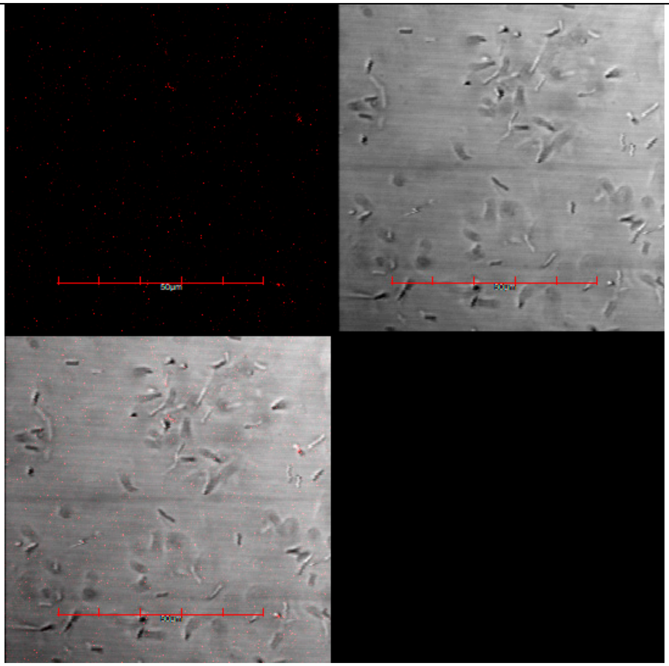	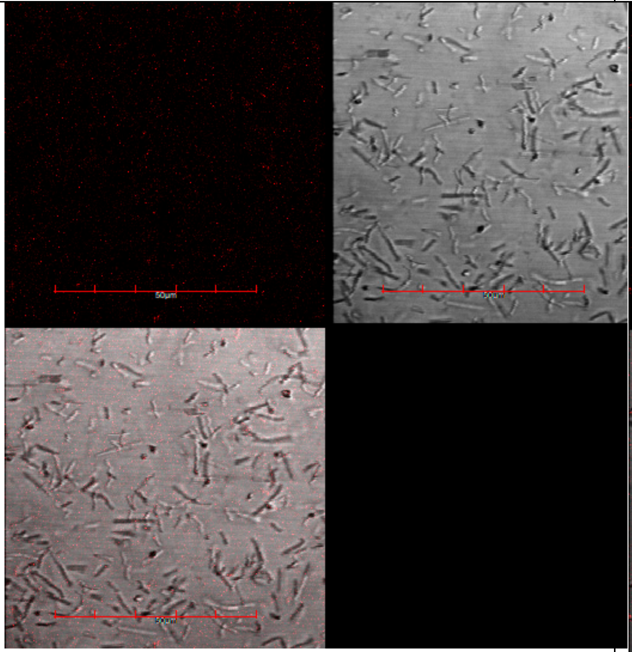	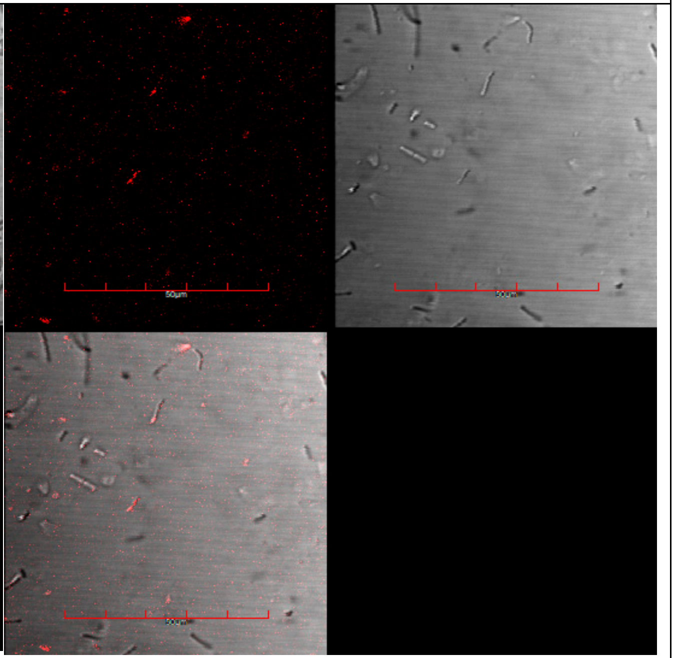

**Dox + EG (only efflux)**	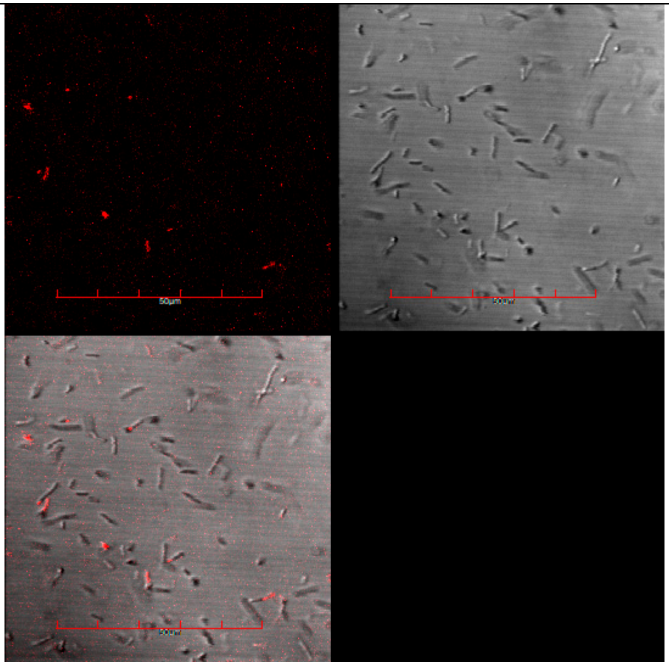	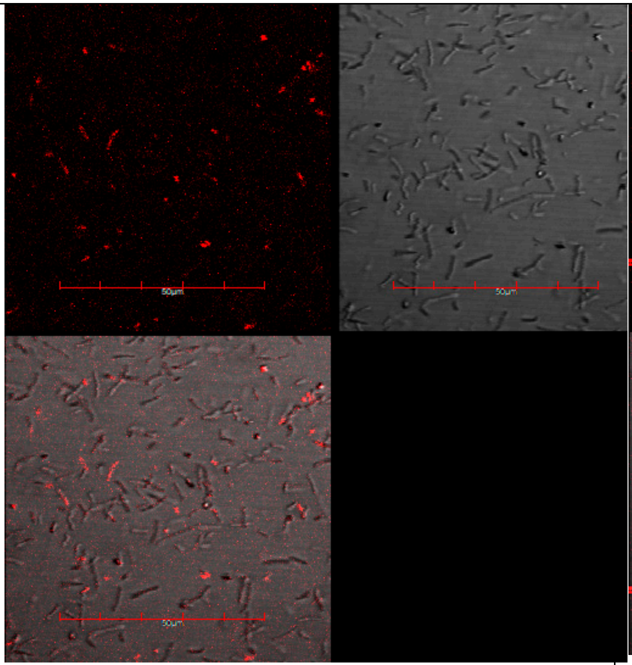	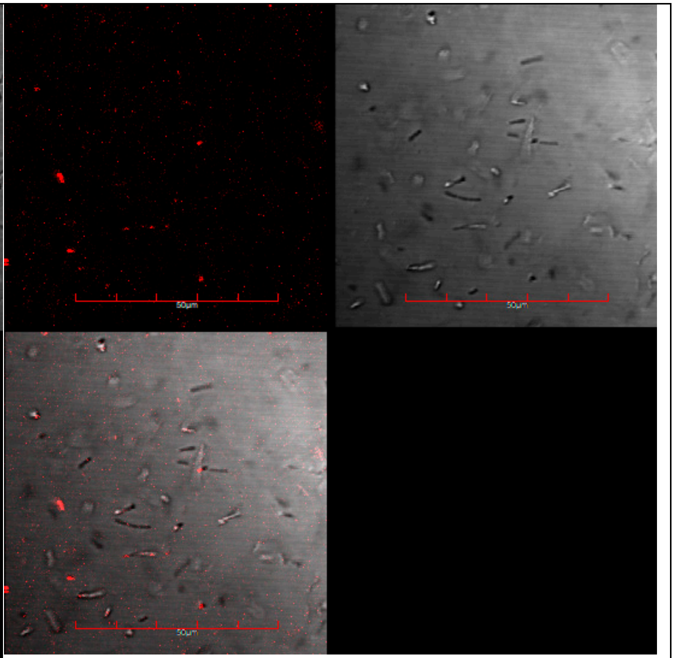
**Dox + FITC-PEI1.8-triMan**	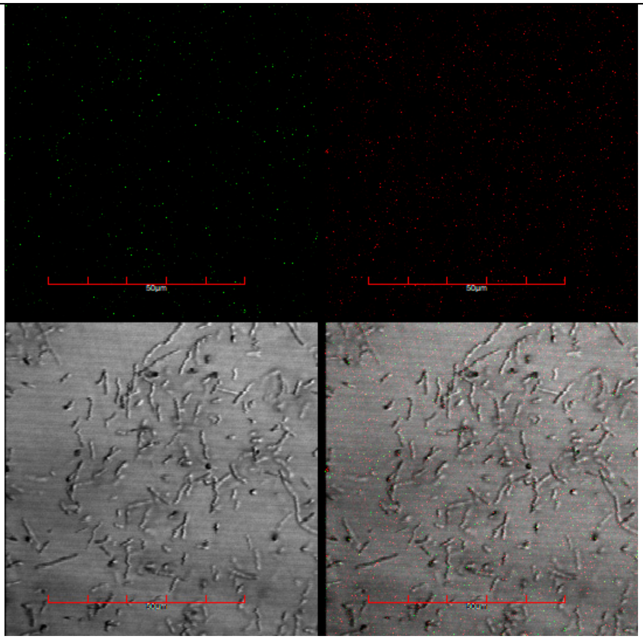	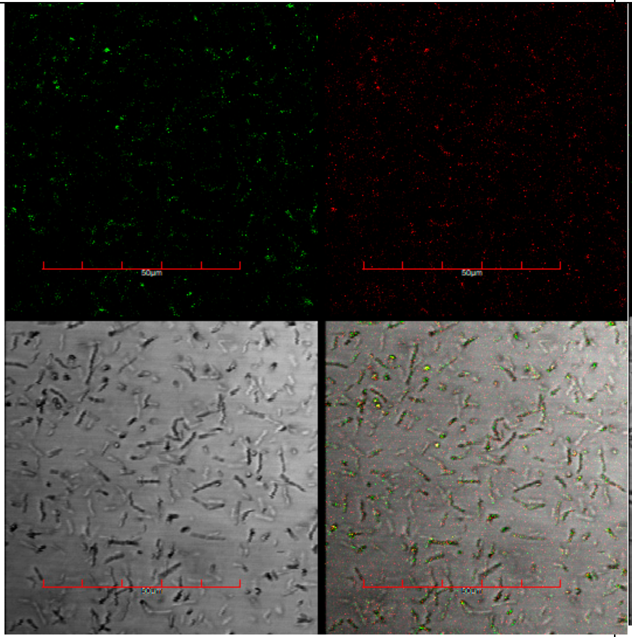	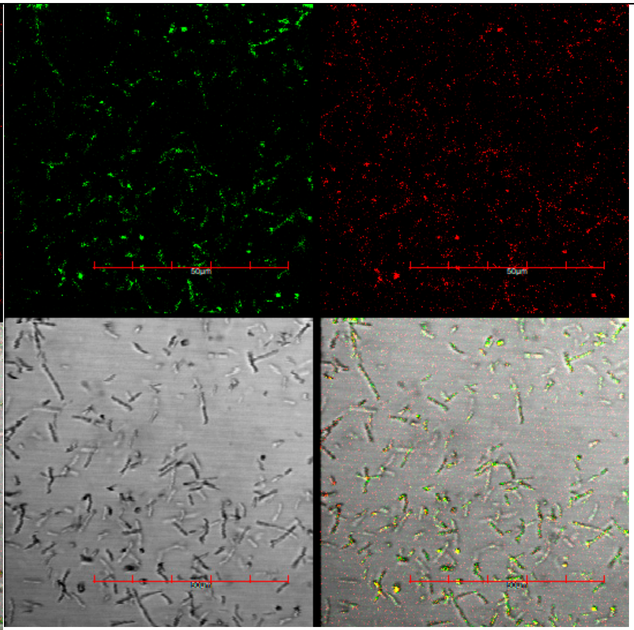

**Dox + FITC-HPCD-PEI1.8-triMan**	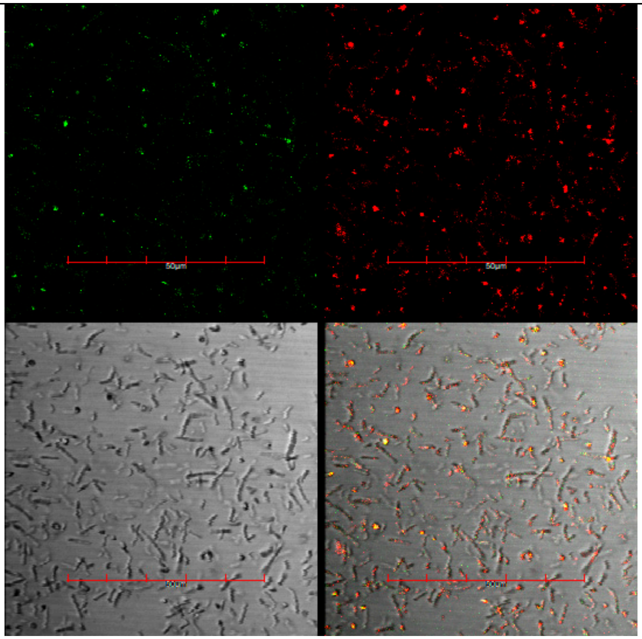	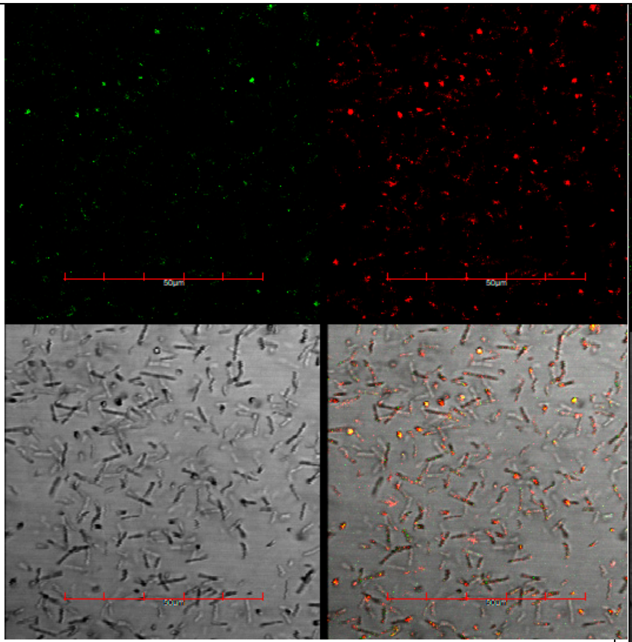	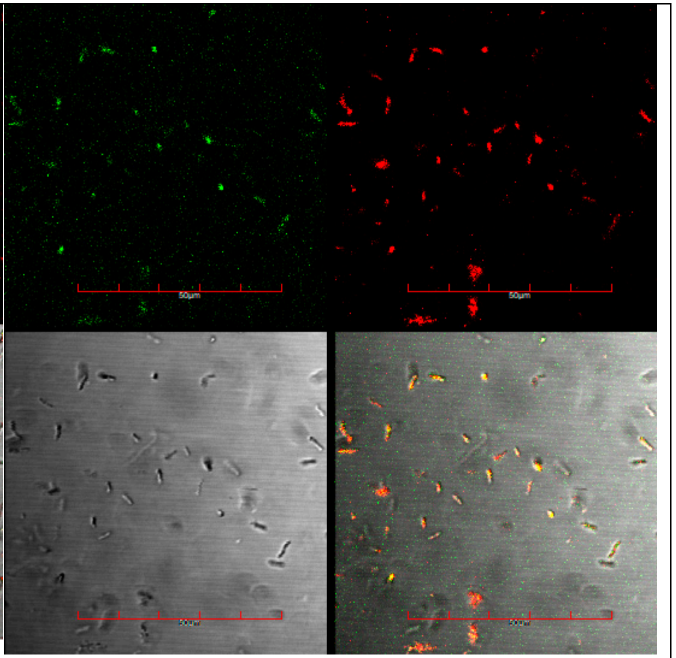
**Dox + EG + FITC-HPCD-PEI1.8-triMan**	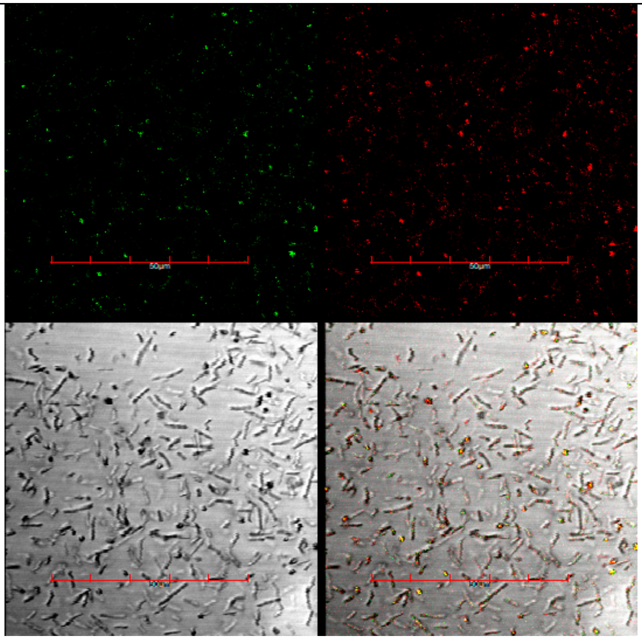	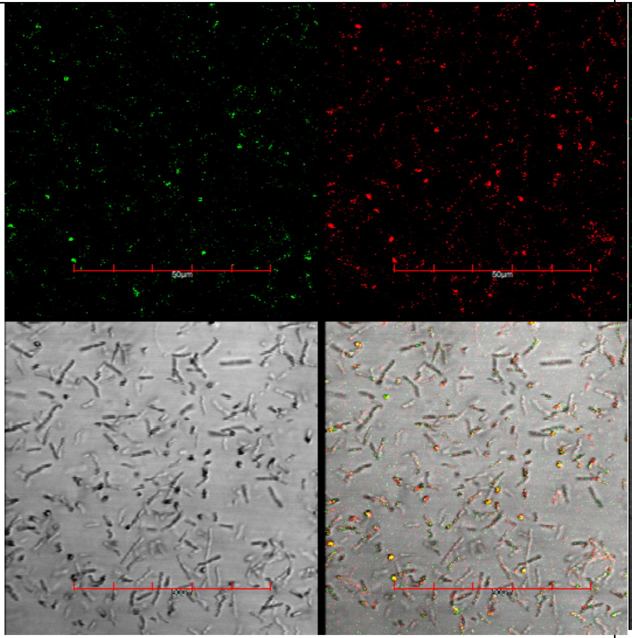	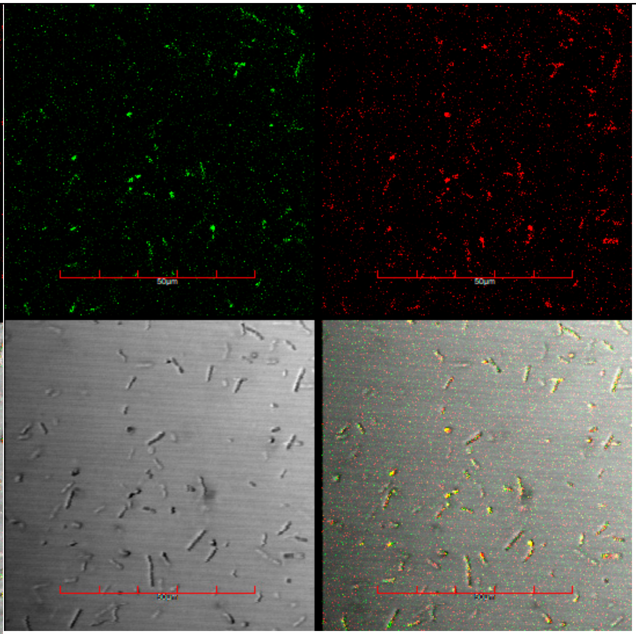

Figure S8. The dependence of *B. subtilis* colony-forming units (CFU) on the incubation time with **(a)** EG (0.1 mg/mL), **(b)** SDS (0.1%). Medium LB (pH 7.2), 37 °C.

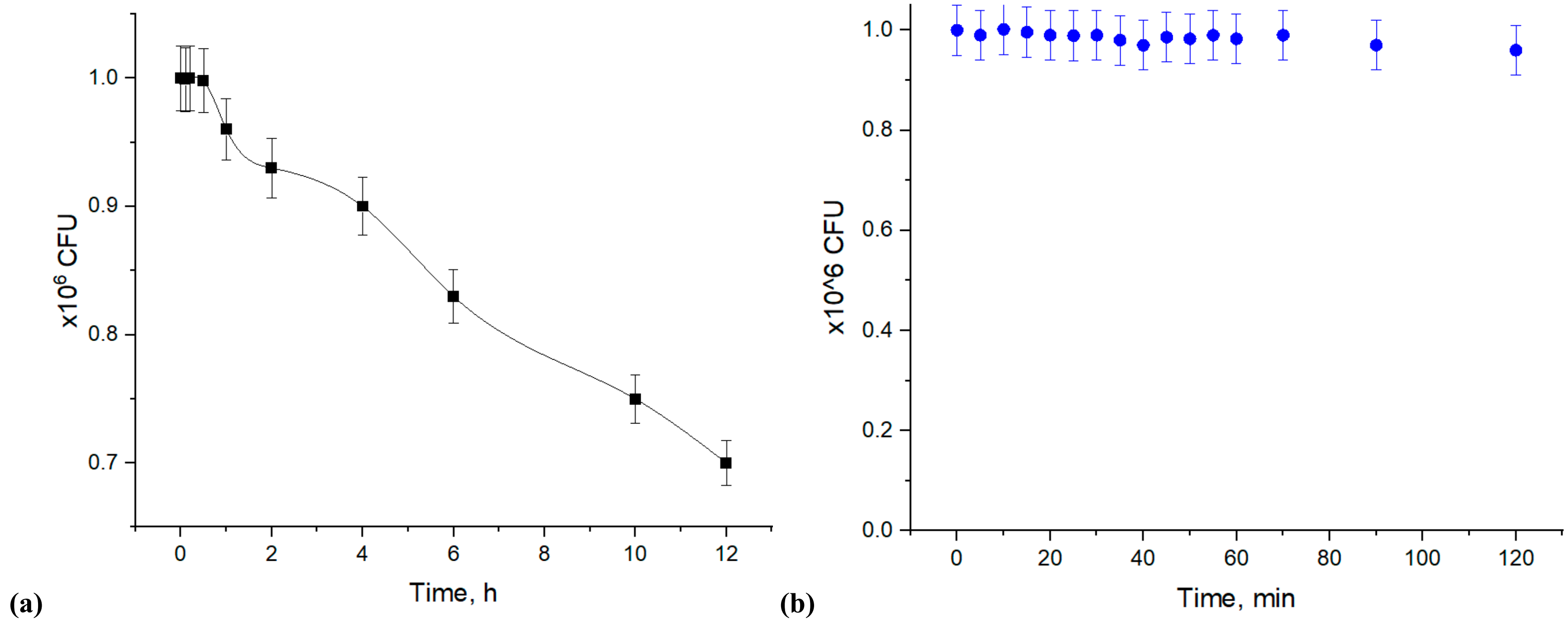

Figure S9. Fourier transform infrared spectra of centrifuged *B. subtilis* followed by resuspension in PBS (0.01 M, pH 7.4): **(a)** cells alone; **(b)** cells hour-incubated with 0.1 mg/mL Dox-MCD and 0.1 mg/mL Dox-MCD with 0.1% SDS. CFU = $3 \cdot 10^6$. T = 22 °C.

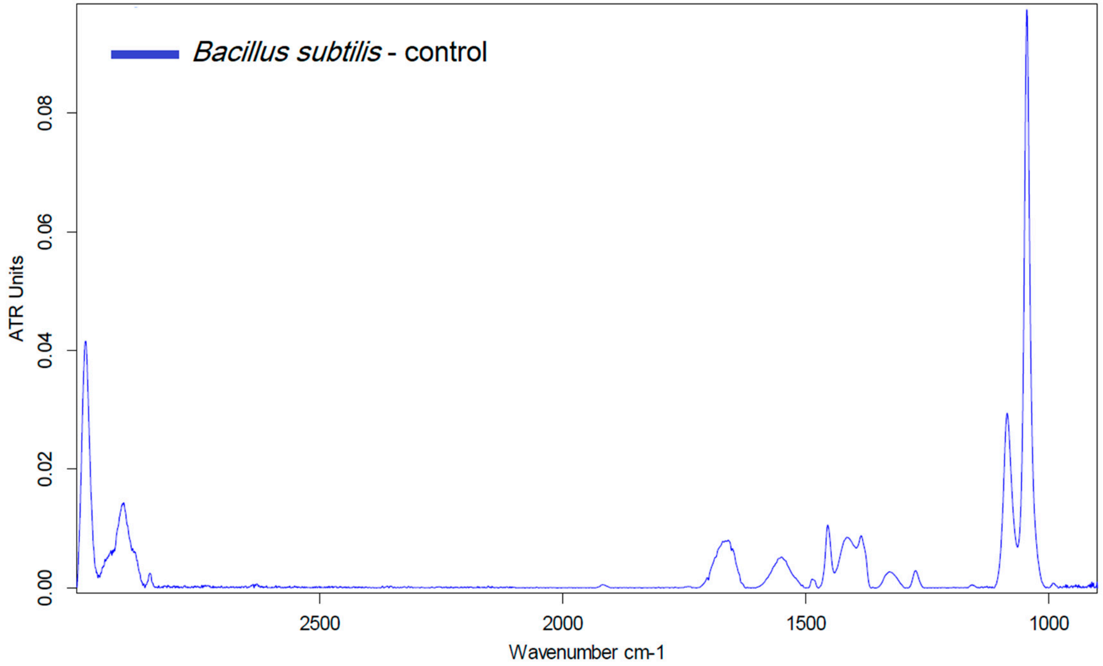

(a)

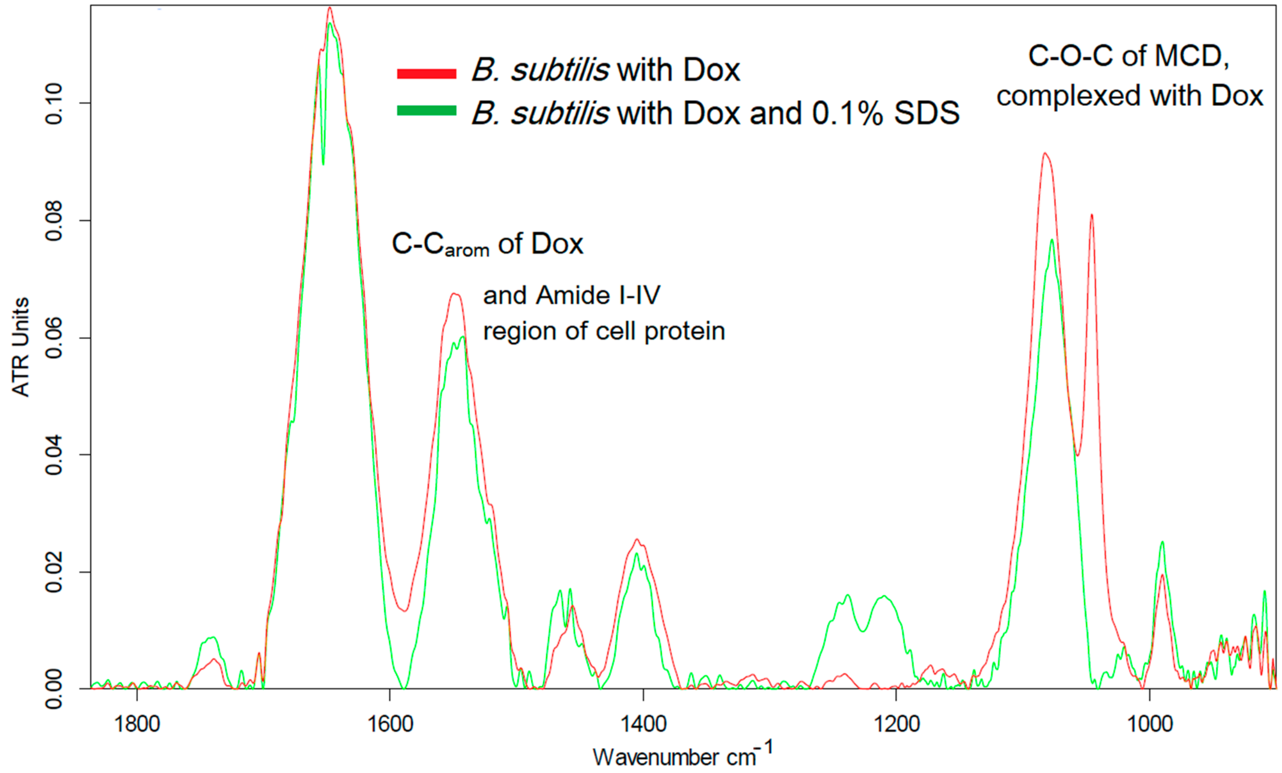
B. subtilis with Dox
B. subtilis with Dox and 0.1% SDS
C-O-C of MCD, complexed with Dox
C-Carom of Dox
and Amide I-IV region of cell protein
ATR Units
0.00
0.02
0.04
0.06
0.08
0.10
1800
1600
1400
1200
1000
Wavenumber cm-1

(b)

Figure S10. Emission fluorescence spectra of FITC and Dox obtaining by CLSM. λ_{em} = 488 nm (multiline Argon laser).

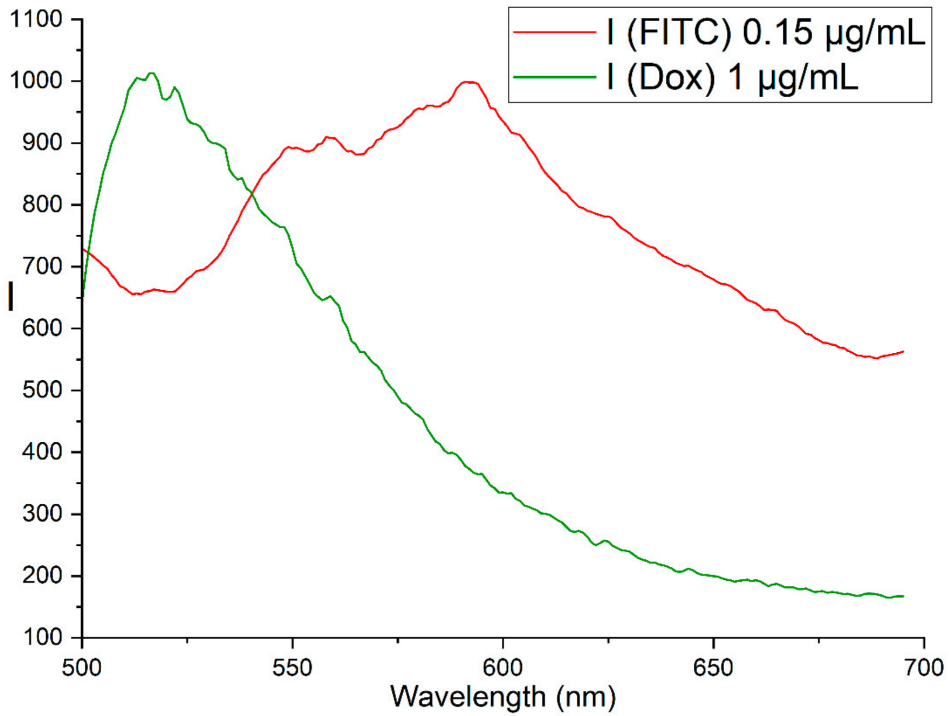

Figure S11. Immunocytochemical evaluation of CD206 in primary culture of human dermal fibroblasts (HDF) (red channel – low intensity). Phagocytosis assay with HPCD-PEI1.8-triMan and HPCD-PEI1.8-Man conjugated with a FITC fluorescent label after incubation for 40 min (green channel). Phase contrast microscopy, fluorescent microscopy, blue channel — nuclei stained with DAPI. Scale bar, 100 µm.

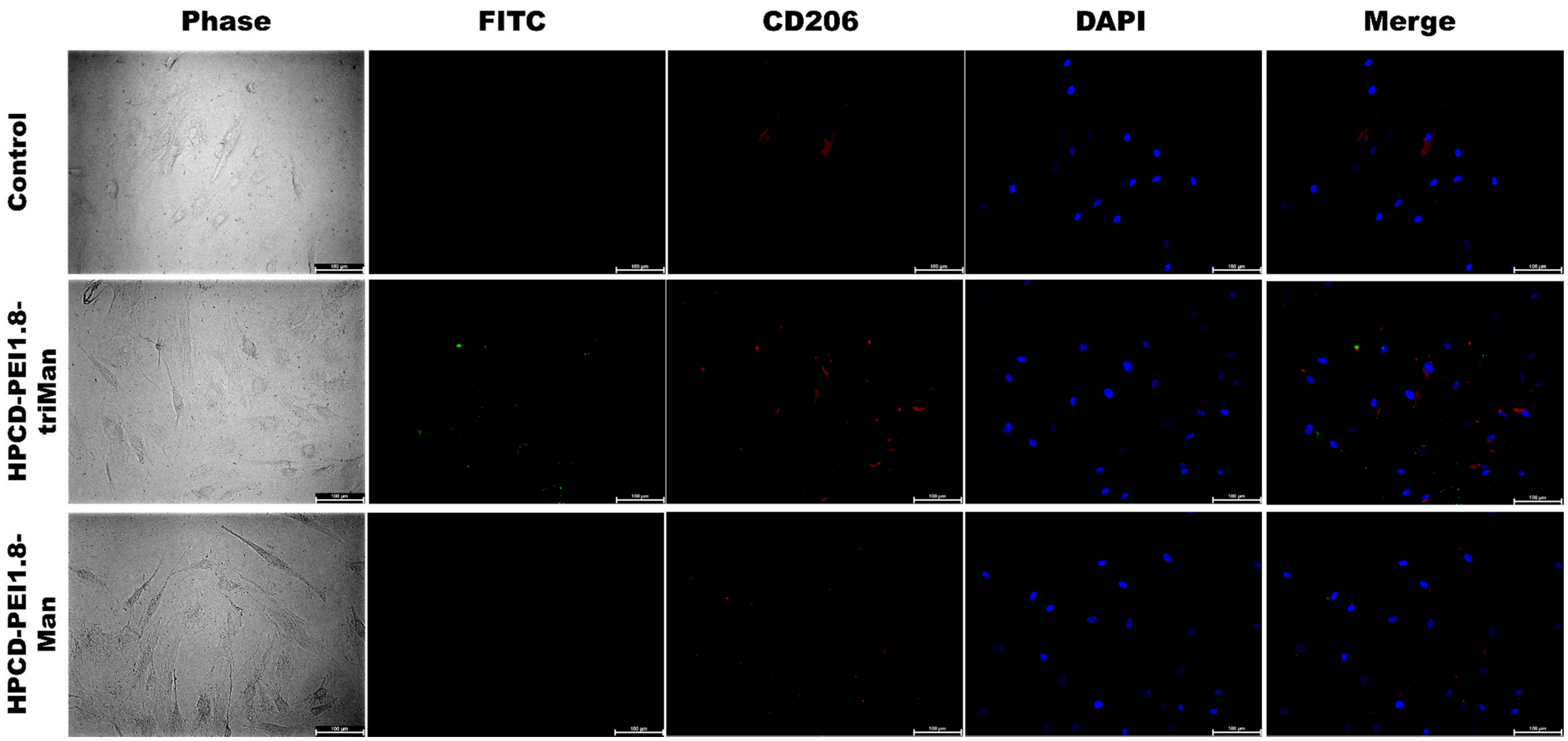

Figure S12. Flow cytometry data on distribution of the number of human dermal fibroblast cells by the intensity of absorbed FITC-labeled conjugates HPCD-PEI1.8-triMan and HPCD-PEI1.8-Man.

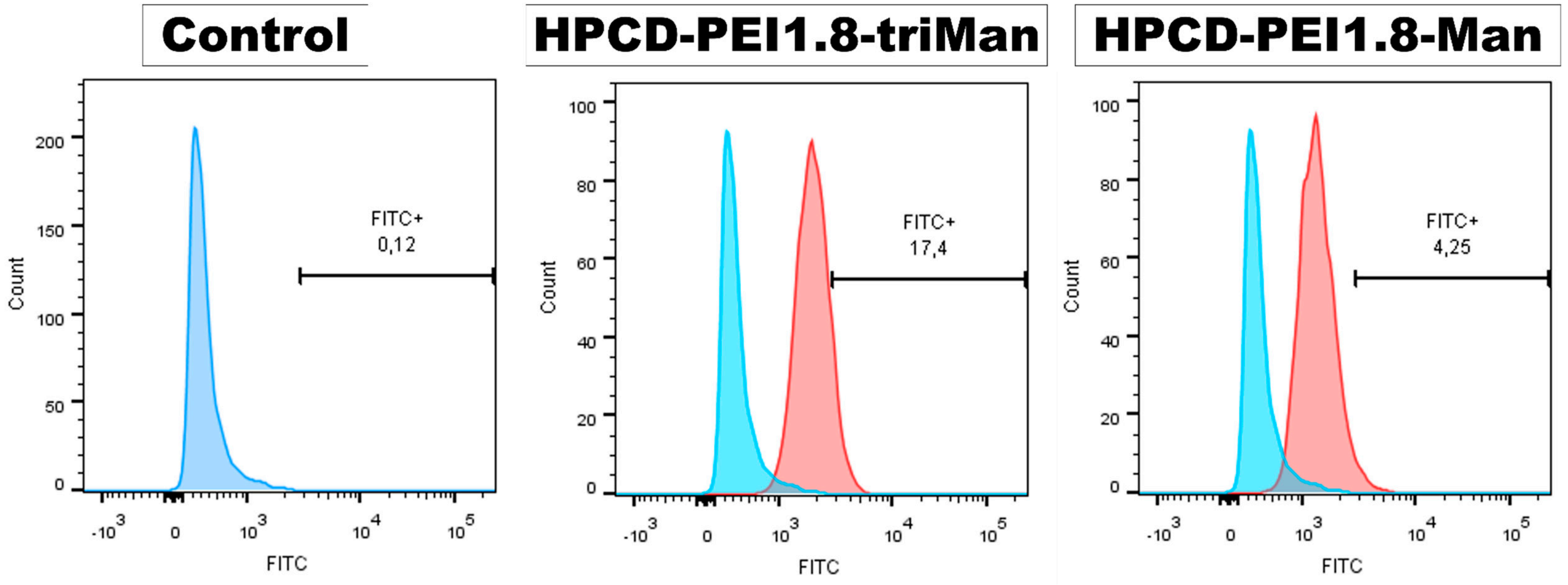

Supplement: Supplementary file 1 [file pharmaceuticals-15-01172-s001.zip › pharmaceuticals-1928511-supplementary.pdf]
